# Resistance Training Improves Sleep and Anti-Inflammatory Parameters in Sarcopenic Older Adults: A Randomized Controlled Trial

**DOI:** 10.3390/ijerph192316322

**Published:** 2022-12-06

**Authors:** Helton de Sá Souza, Camila Maria de Melo, Ronaldo Delmonte Piovezan, Rafael Eduardo Eustórgio Pinheiro Chagas Miranda, Miguel Araujo Carneiro-Junior, Bruno Moreira Silva, Ronaldo Vagner Thomatieli-Santos, Sergio Tufik, Dalva Poyares, Vânia D’Almeida

**Affiliations:** 1 Department of Physical Education, Universidade Federal de Viçosa, Viçosa 36570-900, MG, Brazil; 2 Department of Psychobiology, Universidade Federal de São Paulo, São Paulo 04023-062, SP, Brazil; 3 Department of Nutrition, Universidade Federal de Lavras, Lavras 37200-000, MG, Brazil; 4 Faculty of Health and Medical Sciences, The University of Adelaide, Adelaide 5000, Australia; 5 Department of Physiology, Universidade Federal de São Paulo, São Paulo 04023-062, SP, Brazil; 6 Department of Biosciences, Universidade Federal de São Paulo, Santos 11015-020, SP, Brazil

**Keywords:** sleep quality, physical exercise, aging, skeletal muscle

## Abstract

Sleep and exercise have an important role in the development of several inflammation-related diseases, including sarcopenia. Objective: To investigate the effects of 12 weeks of resistance exercise training on sleep and inflammatory status in sarcopenic patients. Methods: A randomized controlled trial comparing resistance exercise training (RET) with a control (CTL) was conducted. Outcomes were obtained by physical tests, polysomnography, questionnaires, isokinetic/isometric dynamometry tests, and biochemical analysis. Results: Time to sleep onset (sleep latency) was reduced in the RET group compared to the CTL group (16.09 ± 15.21 vs. 29.98 ± 16.09 min; *p* = 0.04) after the intervention. The percentage of slow-wave sleep (N3 sleep) was increased in the RET group (0.70%, CI: 7.27–16.16 vs. −4.90%, CI: 7.06–16.70; *p* = 0.04) in an intention to treat analysis. Apnea/hour was reduced in the RET group (16.82 ± 14.11 vs. 7.37 ± 7.55; *p* = 0.001) and subjective sleep quality was improved compared to the CTL (−1.50; CI: 2.76–6.14 vs. 0.00; CI: 1.67–3.84 *p* = 0.02) in an intention-to-treat analysis. Levels of interleukin-10 (IL-10) (2.13 ± 0.80 vs. 2.51 ± 0.99; *p* < 0.03) and interleukin-1 receptor antagonist (IL-1ra) (0.99 ± 0.10 vs. 0.99 ± 0.10 ng/mL; *p* < 0.04; delta variation) were increased in the RET group. Conclusions: RET improves sleep parameters linked to muscle performance, possibly due to an increase in anti-inflammatory markers in older sarcopenic patients.

## 1. Introduction

Sleep is a behavioral state that is characterized by relative immobility and reduced responsiveness and can be distinguished from coma or anesthesia by its rapid reversibility [1]. Sleep has a number of functions, which include metabolism modulation and the repair of organic tissue [2] There is an increasing body of evidence indicating that sleep debt negatively impacts skeletal muscle trophism [3] and that acute sleep deprivation can lead to muscle atrophy [4], mainly of type II fibers in rats [5,6] in addition to impairing the recovery of injured muscles [7]. Similarly, epidemiological studies have shown that markers of poor sleep are associated with age-related muscular decline and that sleep can play a crucial role in the etiological mechanisms of skeletal muscle disease [8,9].

Furthermore, the aging process is associated with a reduction in several sleep parameters, including total sleep time (TST), the duration of deeper sleep stages, and sleep quality [10]. Moreover, there is an increase in the prevalence of sleep disorders such as insomnia, sleep movement disorders, obstructive apnea syndrome, and sleep–wake disturbances in older adults [11], which can result in chronic sleep debt. This can have a negative impact on a wide range of health factors, including muscle function, which is an important parameter in relation to health status at this stage of life. It should also be noted that there is strong evidence showing that the aging process and sleep debt are directly associated with increased inflammation [12,13]. An increase in inflammatory markers is typically observed in sleep disorders [14,15] and in conditions with reduced muscle function such as sarcopenia [16,17,18].

Sarcopenia is a muscular disease associated with the aging process and is characterized by reduced muscle strength and/or performance (walking speed) and a reduction in muscle quantity [19]. This condition impairs the ability to perform daily living activities, increases the risk of falls and fractures, and is associated with cardiac and respiratory diseases and cognitive impairment. It can lead to mobility disorders, a reduced quality of life, a loss of independence, the need for a long-term care placement, and even death [17]. It should be noted that proinflammatory cytokines increase during muscle wasting, resulting in increased catabolism, and they can also inhibit protein synthesis in skeletal muscle, damaging muscle integrity and function, thereby resulting in sarcopenia [20].

On the other hand, an increase in anti-inflammatory cytokines can antagonize the expression and activity of pro-inflammatory cytokines to reduce muscle atrophy and retard sarcopenia. Some studies suggest that anti-inflammatory cytokines have the potential to be used as therapeutic targets for the treatment of sarcopenia [21].

In this context, it is already known that resistance exercise can improve muscle strength and function, and more recently, it has been suggested that it might also reduce inflammation and improve sleep parameters in different age groups [22,23,24]. According to a recent meta-analysis, resistance training programs that take place more than 3×/week over 12 weeks and that include exercises for at least eight muscle groups can reduce the concentration of inflammatory markers such as IL-6 and TNF-α [25]. In animal models, resistance training has been shown to reduce the deleterious effects of sleep deprivation on skeletal muscle [26]. Although sleep-associated deleterious effects on muscle health are in part a possible explanation for the development and persistence of sarcopenia in older individuals, little is known about the effects of exercise on sleep, especially in the context of sarcopenia. A common thread that connects sarcopenia and reduced sleep quality is the pro-inflammatory status that is frequently observed during the aging process.

Thus, the present study aimed to investigate the effects of a 12-week resistance exercise training (RET) protocol on subjective and objective sleep parameters in older individuals with sarcopenia and the possible role of inflammation status in this process.

## 2. Materials and Methods

### 2.1. Study Design

A randomized, placebo-controlled, parallel-group study testing the efficacy of a 12-week progressive load intervention with resistant exercise was conducted. The protocol was approved by the Ethics Committee of the Universidade Federal de São Paulo (UNIFESP, #51594215.9.0000.5505). This study was conducted according to the ethical standards defined in the 1964 Declaration of Helsinki as well as its subsequent amendments, and it was registered on Clinical Trials (NCT03616249). Written informed consent was obtained from all volunteers.

Candidates were sought through digital, print, and broadcast media. Information about the research and a request for volunteers were disseminated via the institutional website of UNIFESP as well as through different social media. In addition, advertisements were placed in newspapers that are distributed free of charge at subway stations in the city of Sao Paulo. The initial screening of possible participants was undertaken by phone. Individuals aged 65 or over from both genders with no history of current smoking and who did not consume alcohol frequently [27] or use psychoactive drugs were initially invited to participate. Of the 358 subjects contacted by phone, 101 individuals were available to participate in the face-to-face initial evaluation. Thirty-eight individuals with hypertension, diabetes, and hypercholesterolemia not controlled by drugs or with a history of heart failure or who had participated in a physical training program in the last year were excluded. However, volunteers who were only minimally active were included (at least 150 min of physical activity per week assessed by the modified International Physical Activity Questionnaire for the Elderly) [28]. The remaining sixty-three volunteers were referred to the laboratory for blood collection and the initial tests to identify sarcopenia and evaluate sleep parameters. After all the evaluations had been completed, three volunteers were excluded due to recent diagnoses of breast cancer, glaucoma, unstable ischemic heart disease, and eventually, personal reasons.

**Figure 1:**
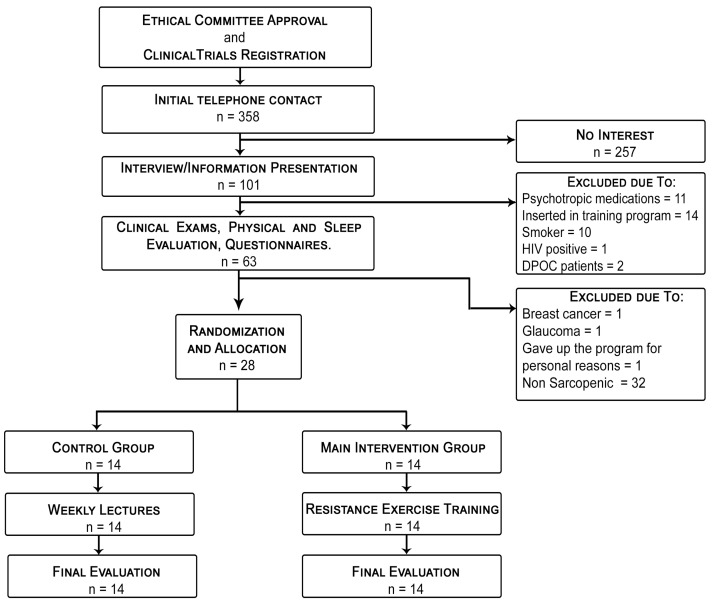
Study flow diagram.

Among the sixty initial candidates, twenty-eight were diagnosed with sarcopenia and were evaluated by polysomnography and serum analyses focused on anabolic hormones and pro- and anti-inflammatory cytokines, performed 24 h before the beginning of the experimental protocol and 24 h after its end. Randomization occurred by using the RANDBETWEEN function (1:1 basis) in Microsoft Excel 365^®^, with a subsequent allocation of the volunteers into two groups: control placebo (CTL, *n* = 14) or resistance exercise training (RET, *n* = 14). The flow diagram of the selection process is presented in Figure 1.

### 2.2. Sarcopenia Identification

The criteria used to identify sarcopenia were previously defined elsewhere and involved reduced muscle strength and performance [19]. Dual Energy X-ray absorptiometry (DXA—GE Healthcare, Madison, WI, USA) was used to analyze body composition and establish the appendicular mass index (AMI). The association of an AMI <7.27 g/m^2^, handgrip strength <40 kg (measured by PC5030JI JAMAR dynamometer, Sammons Preston, Bolingbrook, IL, USA), and/or low physical performance (Short Physical Performance Battery, SPPB-score <6 points) defined sarcopenia in men. The association of an AMI < 5 g/m^2^, handgrip strength <30 kg, and/or low physical performance (SPPB score <6 points) defined sarcopenia in women [19].

### 2.3. Sleep Measures and Definitions

In-lab full-night polysomnography (PSG) was performed using a digital polysomnography system (Embla^®^ N7000, Embla Systems, Inc., Broomfield, CO, USA), applying the standard American Academy of Sleep (AASM) criteria for sleep staging (N1, N2, and N3—non-rapid eye movement—NREM), REM sleep, awakenings, periodic movements of the lower limbs [29], and the number of respiratory events increasing [30]. Recordings began at the patient’s habitual bedtime and finished at 7 a.m. The sleep variables considered were total sleep time (TST), sleep efficiency (SE—the ratio between TST and total recording time × 100), sleep latency (SL), REM latency (LREM), and wake after sleep onset (WASO). Analyses of the events during PSG were conducted by two blinded investigators using international criteria. Apneas were defined as an absence of airflow on the orinasal thermistor or nasal pressure cannula for ≥10 s. Hypopneas were defined as a ≥30% reduction from baseline in airflow for ≥10 s associated with at least a 3% oxygen desaturation or arousal. The AHI was calculated as the mean number of apneas and hypopneas per hour of sleep [31]. To assess subjective sleepiness, sleep quality, and insomnia severity, the Epworth Sleepiness Scale (ESS) [32], the Pittsburgh Sleep Quality Index (PSQI) [33], and the Insomnia Severity Index (ISI) [34] were applied.

### 2.4. Evaluation of Muscular Strength and Torque

Participants initially attended three adaptation sessions on three different days [35,36,37]. At each session, the subjects performed one series of 10–15 repetitions with a minimal load as a warm-up to learn proper positioning in the equipment. The one-maximum repetition (1RM) test was then performed with up to five attempts to achieve maximum load in the training equipment, with 3–5 min of rest between them. The attempts were not classified as valid if the participant did not complete one repetition correctly or completed more than one execution without perfect technique.

Peak torque (PT) was evaluated using the Biodex System 3 dynamometer (Biodex Medical Systems, Shirley, New York, NY, USA). After a 5-min warm-up (stationary bike), subjects were seated on the dynamometer chair, and their body axis was adjusted to the rotation axis of the dynamometer’s input arm. Velcro^®^ (Manchester, NH, USA) straps were used on the thigh, pelvis, and body trunk to stabilize the isolation extension/flexion movements, and gravity force was corrected.

Isokinetic PT was measured in 3 sets of 4 knee extension actions at 60°/s, 45° range of motion with a 30-s interval [38]. The isometric PT test was performed with the knee flexed at 90° with 3 sets of 10 s [39]. All tests were on the non-dominant leg, and for both tests, the volunteers were instructed and encouraged to perform the motion with maximum force (dynamic or static, respectively).

### 2.5. Hormonal, Metabolic and Inflammatory Markers

Fasting venous blood was collected (07:00–10:00 am). A complete blood count was performed (cytochemical/isovolumetric method—Siemens Healthcare^©^, Erlangen, Germany). Serum and plasma aliquots were frozen at −80 °C and analyzed to measure triglycerides, total cholesterol, cholesterol fractions, and glucose (analyzed using Beckman Coulter^Inc.^ colorimetric-enzymatic assays—Beckman Coulter^Inc.^, CA, USA), urea, creatinine, albumin, total testosterone, cortisol (analyzed using chemiluminescence assays—Abbott^®^, IL, USA), growth hormone (GH), insulin-like growth factor 1 (IGF-1), tumor necrosis factor-alpha (TNF-α), interleukin-1 receptor antagonist (IL-1ra), interleukin (IL)-6, and IL-10 using immunoenzymatic assay methods (RayBio^®^ ELISA Kits, Peachtree Corne, GA, USA).

### 2.6. Resistance Training Protocol

**Figure 2:**
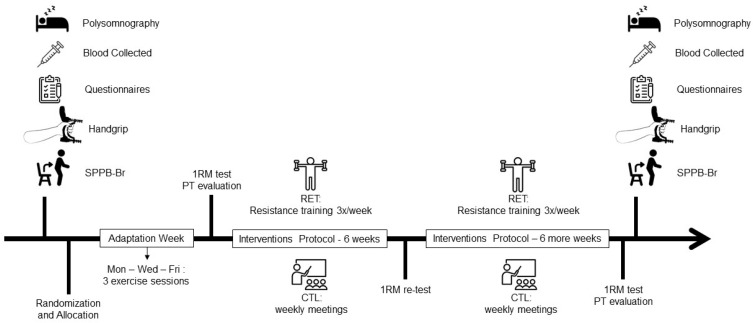
Step study diagram. After recruiting the volunteers, full-night sleep polysomnography was performed. Immediately after waking, blood collection was performed, followed by the application of the questionnaire, the handgrip test, and the physical performance test battery. After randomization, allocation, and adaptation to the tests, 1RM (repetition maximum) and PT (peak torque) were performed. After 6 weeks, a new 1RM test was conducted in the RET group to adjust the training load, and 24 h after the end of the 12 weeks of interventions, 1RM and PT were re-evaluated. Sleep, biochemistry, questionnaire, and physical parameters were tested after 48 h post-training. SPPB-Br: Brazilian version of Short Physical Performance Battery; CTL: control group; RET: exercise training.

The RET followed the current guidelines of the American College of Sports Medicine for the training of older adults [40]. Eight exercises for large muscle groups used the Technogym Selection Pro^®^ (Cesena, Itália) equipment, alternating upper and lower limbs (chest press, leg press, vertical traction, abdominal crunch, leg extension, arm curl, leg curl, and arm extension). RET was performed 3×/week for 12 weeks using a linear periodization model. In the first week, participants performed 1 set of 12 to 15 repetitions at 50% of 1RM. In the second week, they achieved 60% of 1RM, performing 2 sets of 10 to 12 reps. From the third week to the end of the protocol, 75% of 1RM was achieved (3 sets of maximum 8 reps). The intervals between series varied from 60 to 90 s. The training load was readjusted in the 6th training week using the 1RM test [41,42]. The CTL group participated in weekly meetings with recommendations about lifestyle changes. Figure 2 shows the diagram of this study measurements.

### 2.7. Calculation of Sample Size and Statistical Analysis

A required total sample size of 32 individuals was calculated a priori using a repeated measures ANOVA test, with the within-between interaction considered between the two groups, and with a 0.30 effect size, a 0.90 power, and an α err prob. of 0.05 (G*Power software—Universität Kiel, version 3.1.9.2). The data were evaluated in two different ways. To analyze the effect of the inter- and intra-group interventions, a general linear model (GLM) was used. An intention-to-treat analysis (ITT) and an analysis of inter-group intervention responses (the differences between post- and pre-intervention assessments—delta variation) were performed and compared using the Wilcoxon rank-sum test. Additional subgroup analysis by gender was performed, and the results are presented in Supplemental Material S1. The GLM results were presented as mean ± standard deviation, and for ITT, the results were presented as median with 95% confidence interval (CI) values. For all comparisons, 0.05 was assumed as a significant level (two-sided test), and the software used was Statistic (TIBCO Software Inc. 13.5.0.17) and STATA (StataCorp., Release 14, College Station, TX, USA) software.

## 3. Results

The baseline characteristics of the participants are described in Table 1. The mean ages of participants were 74.6 ± 7.1 years and 77.4 ± 6.2 years for the CTL and RET groups, respectively, with most of them being classified as normal weight according to their body mass index (BMI) and lower appendicular mass index. Males in the CTL and RET groups had higher body mass (Supplementary Material—Table S1). According to criteria established by the AASM, the sample showed increased average sleep latency (CTL: 21.6 ± 15.7 min and RET: 25.9 ± 20.2 min) and N1 sleep stage (CTL: 10.3 ± 4.4% and RET: 20.6 ± 15.8%). Both groups showed, on average, moderate sleep apnea at baseline, with an apnea–hypopnea index of 15.20 ± 4.37 in the CTL group and 14.08 ± 15.59 in the RET group. In addition, the RET group presented an elevated insomnia severity index at baseline (8.71 ± 4.37). Regarding hormonal and inflammatory markers, the participants showed, on average, adequate testosterone, GH, and cortisol levels; reduced levels of IGF-1; and increased levels of IL-6 and IL-10, two anti-inflammatory cytokines. 

### 3.1. Effects of RET on Sarcopenia Parameter Outcomes

**Figure 3:**
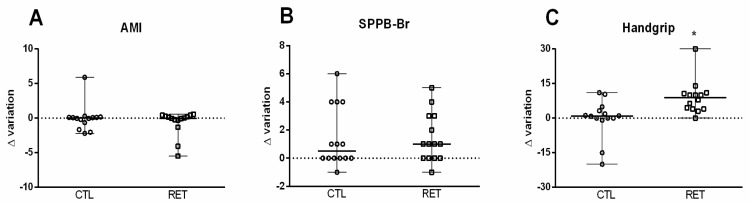
Sarcopenia criteria analyses. (**A**)—AMI: Delta variation appendicular muscle index. (**B**)—Delta variation handgrip strength values. (**C**)—Delta variation SPPB: Short Physical Performance Battery values. CTL: control; RET: resistance exercise training. * Different from CTL, *p* < 0.05.

Considering the results obtained for AMI (Figure 3A) and SPPB (Figure 3B) in the ITT analysis, there were no differences between groups (*p* > 0.05). For handgrip strength (Figure 3C), in the ITT analysis, the RET group presented increased strength (8.95 kg, CI: 5.20–11.57) compared to the CTL group (0.91 kg, CI: 6.02–13.38; *p* < 0.01) in both males and females compared to males and females in the CTL group (Supplementary Material—Table S2). Strength, evaluated using the 1RM test, was greater at both the 6th and 12th weeks in the RET group compared to baseline (Supplementary Material).

### 3.2. Effects of RET on Objective and Subjective Sleep Parameter Outcomes

No differences were observed between the CTL and RET groups for LREM, TST, N2, WASO, and the number of apneas per hour at baseline (*p* = 0.05—Table 2). After the 12-week intervention, there was a reduction in sleep latency in the RET group (16.09 ± 15.21 min) compared to the CTL group (29.98 ± 16.09 min; *p* < 0.04). The CTL group increased delta variation for SL, while in the RET group it decreased (5.25; CI: 9.84–21.87 vs. −6.25; CI: 8.00–17.78; *p* < 0.04). Among the 14 subjects evaluated in the RET group, only two did not have reduced SL.

In respect of sleep stages, the ITT analysis showed that the RET group presented a higher amount of N3 sleep stage (0.70%, CI: 7.27–16.16) compared to the CTL group (−4.90%, CI: 7.06–16.70; *p* < 0.04—Table 2). The majority of individuals in the CTL group presented a reduction in N3 at pre- and post- intervention (32.18 ± 11.57 vs. 27.80 ± 9.08), whereas in the RET group there was an increase or maintenance of this sleep stage (25.55 ± 9.79 vs. 30.25 ± 11.23), possibly no difference between them. For the apnea/hour parameter, although there were no statistical differences between groups (*p* > 0.05), the RET group showed a reduction post-intervention (16.82 ± 14.11 vs. 7.37 ± 7.55; *p* < 0.001), while there were no differences in the same comparison in the CTL group (9.68 ± 4.52 vs. 13.29 ± 7.66; *p* > 0.05—Table 2). For gender comparison, both the males and females in the RET group had a reduced apnea/hour compared to the CTL group (Supplementary Material—Table S3). However, RET reduced AHI (9.37 ± 13.70 n°/h) when compared to CTL (15.13 ± 15.89 n°/h) after 12 weeks of intervention (*p* < 0.01), and only RET females showed the same reduction (Supplementary Material—Table S3).

The insomnia severity was reduced in the RET group after 12 weeks when compared to baseline values (8.71 ± 4.37 vs. 5.14 ± 3.73; *p* < 0.01), but there was no difference (*p* > 0.05) between pre- and post-intervention in the CTL group (5.71 ± 3.89 vs. 5.78 ± 3.78). The RET group presented a reduction in the Pittsburgh delta variation score compared to the CLT group (−1.50; CI: 2.76–6.14 vs. 0.00; CI: 1.67–3.84; *p* < 0.02). Subjective sleep efficiency also improved after 12 weeks in the RET group in comparison to their baseline score (83.57 ± 14.56 vs. 75.57 ± 15.37), and delta variation was different between the RET and CTL groups (9.50; CI: 9.87–21.94 vs. 0.00; CI: 8.04–17.87; *p* < 0.05—Table 3).

### 3.3. Effects of RET on Skeletal Muscle Strength

**Figure 4:**
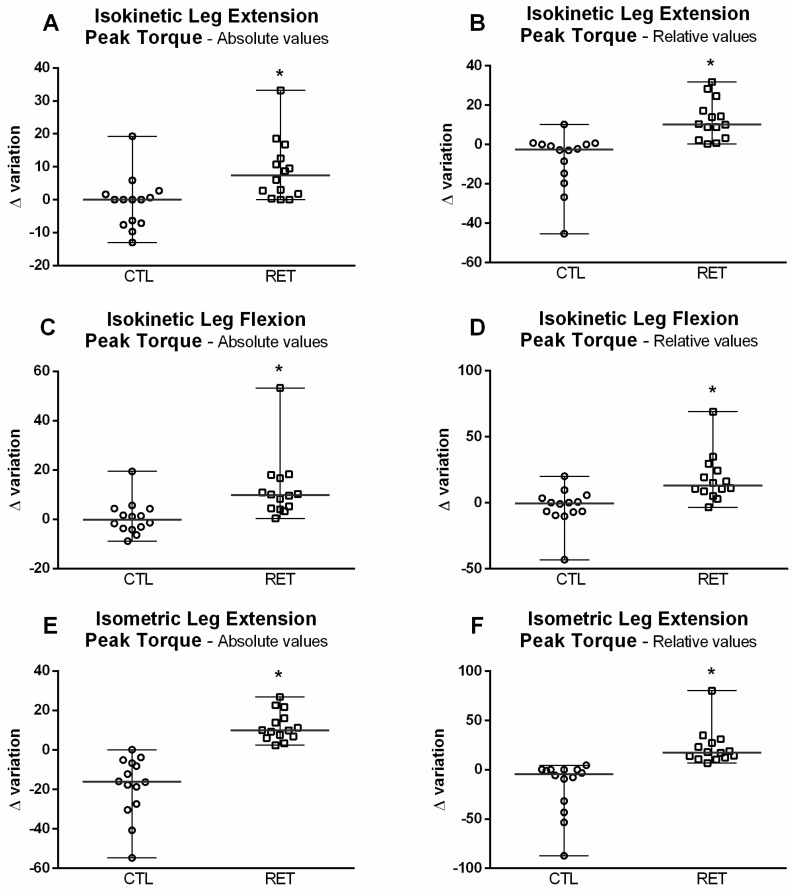
Isokinetic/isometric PT evaluation. (**A**)—Delta variation isokinetic leg extension PT values. (**B**)—Delta variation isokinetic leg extension PT/body mass values; (**C**)—Delta variation isokinetic leg flexion PT values. (**D**)—delta variation isokinetic leg flexion PT/body mass values. (**E**)—delta variation isometric leg extension PT values. (**F**)—delta variation isometric leg extension PT/body mass values. PT: Peak torque. * Different from CTL, *p* < 0.05.

Figure 4 shows that in the RET group there were increases in absolute (7.35 N-M, CI: 6.74–14.98) and relative (10.20%, CI: 7.26–16.14) PT values post-intervention compared to the CTL group (0.00 N-M, CI: 6.74–14.98, *p* < 0.002 and −2.50%, CI: 10.38–23.08, *p* < 0.001, respectively). The results were similar for absolute and relative leg flexion PT, with the RET group values being higher than those of the CTL group in both conditions (absolute flexion PT RET group: 9.80 N-M, CI: 9.44–20.97 vs. CTL: −0.15 N-M, CI: 4.96–11.03, *p* < 0.001; and relative flexion PT RET group: 13.00%, CI: 12.98–28.85 vs. CTL: −0.50%, CI: 10.24–22.77, *p* < 0.001—Figure 4A–D).

Very similar results were also observed in respect of leg extension isometric action (Figure 4E,F). The delta variation of the RET group (9.95N-M, CI: 5.37–11.94) for absolute isometric PT values was higher than the CTL group (−16.15N-M, CI: 11.12–24.72; *p* < 0.001), and the isometric PT relative to body mass was also higher for the RET group (17.45%, CI: 13.37–29.71) compared to the CTL group (−4.50%, CI: 19.61–43.59; *p* < 0.001).

### 3.4. Changes in Hormonal, Metabolic and Inflammatory Marker Outcomes after the RET Intervention

Males in the CTL and RET groups had higher testosterone concentrations (Supplementary Material—Table S4). No differences were found between groups pre- and post-intervention in respect of total testosterone, GH, IGF-1, cortisol, IL-6, IL-10, and TNF-α (Table 4), but there was a reduction in TNF-a in the RET males compared to the CTL-males (Supplementary Material—Table S4). IL-10 increased in the RET group after the 12-week intervention (2.13 ± 0.80 vs. 2.51 ± 0.99; *p* < 0.03) but did not change in the CTL group (1.96 ± 0.58 vs. 2.26 ± 0.88; *p* > 0.09). IL-1ra increased after the intervention when comparing RET and CLT at the same time (0.99 ± 0.10 vs. 0.99 ± 0.10 ng/mL; *p* < 0.04—Table 4) and delta variation was higher in the RET group (0.04 ng/mL, CI: 0.06–0.15) compared to the CTL (−0.01 ng/mL, CI: 0.06–0.15; *p* < 0.05), and for gender comparison, this increase was observed only in the RET-males post-intervention.

## 4. Discussion

This study focused on investigating the effects of RET on the sleep parameters of sarcopenic older adults and the possible role of inflammation status in this process. Although previous research has suggested sleep deprivation can affect muscle trophism in humans, this has not yet been investigated [3,7]. Furthermore, no previous studies have assessed a wide range of subjective and objective sleep parameters in older people diagnosed with sarcopenia. Investigating this area is important, as it has been suggested that there is a potential bidirectional relationship between sarcopenia development and age-related sleep alterations [9]. To the best of our knowledge, this is the first study that sought to objectively assess the effects of resistance training on sleep in sarcopenic older individuals. The results of this randomized controlled trial showed that a 12-week RET protocol simultaneously improved muscle strength and objectively measured sleep quality by reducing sleep latency, increasing NREM stage 3 (N3) sleep, and improving subjective sleep perception. A similar anti-inflammatory pathway can explain both the improved muscle health and sleep quality through the detected increases in IL-10 and IL1-ra [43,44]. However, stratified analysis by gender did not maintain similar results in both genders, possibly due to a lack of statistical power for subgroup analysis.

The European Working Group on Sarcopenia in Older People (EWGSOP [17]) recommends that therapeutic approaches targeting sarcopenia treatment should aim to restore muscle strength since its reduction is the central element of this disease [17]. Therefore, exercise programs, especially RET, are highly recommended for this population [45,46,47,48]. We demonstrated that a RET protocol improved all muscle strength markers (handgrip and 1RM test) and increased peak torque in concentric, isokinetic, and isometric muscular actions. The evaluation of muscular function using an isokinetic dynamometer is one of the most effective ways of measuring strength [49,50]. Muscle strength is an important predictor of physical limitations and disability [51], while an increase in muscle strength, even without an increase in muscle mass, is associated with good physical function. However, we did not identify improvements in SPPB-Br scores. This may be explained by the fact that it is more usual to identify improvements in balance and walk speed following power training protocols rather than traditional resistance training, as applied in our study [52,53].

Regarding sleep parameters, important changes were observed in response to RET in our study. These changes corroborate the findings described by Syed-Abdul and colleagues [24], who observed improvements in sleep associated with increased handgrip strength. In addition to the increase in overall subjective sleep quality, there was also a reduction in sleep latency, AHI, and insomnia severity, as well as an increase in deeper stage 3 sleep in the RET group in comparison with the CTL group. Previous studies have shown that RET programs for general older populations are associated with benefits across a broad range of sleep parameters, including, in respect of, a reduction in sleep latency and sleep fragmentation and an increase in total sleep time (TST) [54]. Although we did not observe an increase in TST or a decrease in sleep fragmentation, our protocol resulted in a decrease in SL and an increase in the proportion of N3 of sleep stage, a stage of deep non-REM sleep. The differences between our data and the findings of previous studies may have been due to the sarcopenic condition of our volunteers.

Some authors have suggested that N3 and REM sleep are associated with the greater release of growth hormone (GH) and testosterone, respectively, while sleep debt increases cortisol concentrations [55]. Until now, these were the bases that supported the relationship between sleep debt and muscle trophism [3]. Although we did not observe changes in GH and testosterone or any change in sleep architecture in the RET group, we found higher IL-10 and IL-1ra concentrations. Thus, unlike what was proposed in the studies by Dáttilo et al. [3] and Monico-Neto et al. [56], it is possible that the relationship between sleep and skeletal muscle does not occur through endocrine alterations generated by the sleep debt, but rather that changes in sleep parameters stimulate the phenomenon known as anabolic resistance [5], which contributes to the development of sarcopenia, and through a mechanism that has not yet been fully elucidated, RET improves anabolic sensitivity, resulting in improved muscle function.

Although no increase in anabolic hormones or decrease in catabolic hormones were observed, muscle hypertrophy and increased strength may occur regardless of hormone levels [57]. Mechanical overload, local metabolites, or intermittent hypoxia generated by muscular actions may contribute to improved muscle integrity, increased signaling for muscle fiber accumulation, and increased muscle strength [57].

Previous studies focusing on protocols of acute sleep deprivation or restriction have shown that both can promote pro-inflammatory properties [58,59,60]. Acute sleep debt, similar to other stress conditions, alters the activity of the hypothalamic-pituitary-adrenal axis (HPA) [60]. An increase in HPA axis activity stimulates the acute phase response, including the activation of toll-like receptors (TLRs), a class of receptors that recognize molecular patterns of self and non-self-microorganisms. TLRs and receptor complexes activate gene transcription that stimulates nuclear factor kappa-beta (NF-kB) and leads to the production of a class of proteins called inflammatory cytokines, such as IL-1β, TNF-α, and IL-6. Furthermore, this immune cascade also activates phagocytic cells, such as neutrophils, monocytes, and macrophages, which can also increase the production of these inflammatory cytokines [61,62]. IL-1, in particular, has been postulated as an important somnogenic factor [63] associated with poor sleep, increased sleep latency, daytime sleepiness, fatigue [64,65,66], and obstructive sleep apnea syndrome [67]. In addition, IL-1 is associated with circadian disruption [63] and skeletal muscle reduction [68]. Continuous exercise programs can help to reduce inflammatory status [69,70], including by increasing levels of IL-1 receptor antagonists, which are important inhibitors of IL-1 activity, optimizing cellular repair [71].

Therefore, in older adults, inflammatory mechanisms play an important role in the pathophysiology of sarcopenia [72], and sleep disorders seem to contribute to the development of sarcopenia [8]. The improvements in sleep latency and the subjective perception of sleep found in this study indicated that the sleep patterns of sarcopenic individuals were positively affected by RET. Moreover, reductions in sleep latency and insomnia severity have been considered markers of improvement in patients with insomnia symptoms [73]. Therefore, our results indicate improved subjective sleep quality [33] in sarcopenic older adults following the RET program, which may be due to an increase in the concentration of IL-1-ra [74]. In addition, physical exercise promotes an increase in body temperature, central nervous system fatigue, and heart rate, which are all signals that, via feedback mechanisms, can favor the onset of sleep and make it more consolidated [55,75], which, in turn, can improve muscle metabolism.

This study has some limitations. The small sample size combined with the high interindividual variability in the findings increases the probability that some possible differences in other sleep parameters were not demonstrated due to type II error. We also did not control the food intake, a factor that can change with increasing exercise levels or screen time in/or before bed, which could affect sleep onset parameters. Moreover, an unblinded protocol, which is methodologically expected once physical activity interventions are applied, increases the risk of bias in the results. On the other hand, the trial has some important strengths, including the use of objective measurements that are considered the gold standard for both sleep (PSG) and muscle evaluation (isokinetic dynamometry and DXA). It is also important to mention that this study is one of the first to study the effects of RET on sleep parameters in sarcopenic older individuals and the difficulties in developing a training protocol in this population.

## 5. Conclusions

In conclusion, a 12-week progressive load resistance training program performed by older adults with sarcopenia simultaneously improved muscle performance, objective and subjective sleep quality, as well as inflammatory status. Future studies are necessary to elucidate how different age groups and genders, with and without sarcopenia, can present specific muscle and sleep responses to potentially anti-inflammatory interventions, such as physical exercise.

## Figures and Tables

**Table 1:** Sample characterization at baseline.

Variables/Groups	CTL	RET
Age _(years)_	74.64 ± 7.13	77.42 ± 6.25
Height _(cm)_	155.17 ± 11.45	162.04 ± 12.81
Body Mass _(kg)_	64.93 ± 14.94	67.63 ± 12.77
Body Fat _(%)_	42.10 ± 6.85	35.62 ± 10.07
AMI _(kg/m^2^)_	6.58 ± 0.88	7.20 ± 2.54
Body Mass Index _(kg/m^2^)_	26.78 ± 4.44	25.54 ± 2.04
Hemoglobin _(g/dL)_	14.04 ± 1.73	14.50 ± 1.15
Hematocrit _(%)_	41.56 ± 5.50	43.77 ± 3.63
Platelets _(thousands/mm^3^)_	192.20 ± 30.84	228.00 ± 62.07
Leukocytes _(thousands/mm^3^)_	5.14 ± 0.92	6.46 ± 1.78
Neutrophils _(thousands/mm^3^)_	3.14 ± 0.60	3.84 ± 1.43
Eosinophils _(thousands/mm^3^)_	0.08 ± 0.07	0.25 ± 0.042
Basophils _(thousands/mm^3^)_	0.03 ± 0.02	0.03 ± 0.01
Typical lymphocytes _(thousands/mm^3^)_	1.57 ± 0.44	1.90 ± 0.60
Total lymphocytes _(thousands/mm^3^)_	1.57 ± 0.44	1.90 ± 0.60
Monocytes _(thousands/mm^3^)_	0.30 ± 0.06	0.42 ± 0.18
Cholesterol _(mg/dL)_	171.80 ± 28.98	187.09 ± 42.42
HDL _(mg/dL)_	63.40 ± 18.18	54.63 ± 10.82
NO HDL _(mg/dL)_	108.40 ± 15.10	132.45 ± 41.12
LDL _(mg/dL)_	88.20 ± 19.13	109.72 ± 36.33
VLDL _(mg/dL)_	20.20 ± 5.40	22.72 ± 8.05
Triglycerides _(mg/dL)_	101.20 ± 27.72	114.27 ± 40.48
Glucose _(mg/dL)_	99.60 ± 6.61	94.63 ± 12.17
Urea _(mg/dL)_	32.20 ± 3.11	40.90 ± 11.86
Creatinine _(mg/dL)_	0.83 ± 0.08	0.83 ± 0.18
Albumin _(g/dL)_	4.34 ± 0.24	4.24 ± 0.29

Statistical analysis by Generalized Linear Model (GLM—data presented as mean ± standard deviation) with Duncan’s post hoc. CTL = Control group, RET = Resistance Exercise Training group. AMI = appendicular mass index. g/m^2^ = gram/square meter. Kg = kilogram/square meter. g/dL = gram/deciliter. thousand/mm^3^ = thousand units/cubic millimeter. mg/dL = milligram/deciliter. % = percentage.

**Table 2:** Evaluation of sleep aspects through full-night polysomnography.

Variables/Groups	CTL				RET			
	Baseline	After 12 Weeks	∆ Variation		Baseline	After 12 Weeks	∆ Variation	
			Median	95%CI			Median	95%CI
Sleep Latency _(min)_	21.59 ± 15.67	29.98 ± 16.09	5.25	9.84–21.87	25.86 ± 20.16	16.09 ± 15.21 *	−6.25 ^#^	8.00–17.78
REM sleep latency _(min)_	86.21 ± 44.34	103.00 ± 45.42	9.00	29.32–65.15	108.53 ± 73.17	117.10 ± 70.87	0.25	55.69–123.76
Total Sleep Time _(min)_	342.00 ± 90.03	325.14 ± 66.56	−28.00	43.34–96.31	310.00 ± 61.17	298.86 ± 84.93	8.50	46.72–103.84
Sleep Efficiency _(%)_	72.32 ± 18.74	69.49 ± 13.31	−3.80	10.60–23.57	67.05 ± 13.52	67.92 ± 20.07	1.70	12.26–27.26
N1 _(%)_	10.27 ± 4.39	10.23 ± 3.85	0.80	3.79–8.42	20.62 ± 15.79	22.31 ± 21.34 *	−0.15	12.27–27.28
N2 _(%)_	39.57 ± 11.79	42.60 ± 10.63	3.20	4.73–10.52	37.34 ± 11.95	37.37 ± 8.66	−1.80	9.70–21.57
N3 _(%)_	32.18 ± 11.57	27.80 ± 9.08	−4.90	7.06–15.70	25.55 ± 9.79	30.25 ± 11.23	0.70 ^#^	7.27–16.16
WASO _(min)_	80.21 ± 46.55	98.63 ± 40.77	17.30	36.04–80.10	128.42 ± 58.21	131.21 ± 94.95	9.05	61.99–137.76
Apnea/hour _(n°/h)_	9.68 ± 4.52	13.29 ± 7.66	1.25	5.56–12.37	16.82 ± 14.11	7.37 ± 7.55 ^†^	−9.45	6.93–15.41
AHI _(n°/h)_	15.20 ± 13.09	15.13 ± 15.89	12.35	4.91–10.93	14.08 ± 15.59	9.37 ± 13.70 *	9.90	5.34–11.88
SPO2 < 90% _(min)_	7.02 ± 10.67	6.55 ± 11.03	0.00	4.74–10.54	6.84 ± 10.29	5.00 ± 8.67	−0.85	2.04–4.53

Statistical analysis by Generalized Linear Model (GLM–data presented as mean ± standard deviation) with Duncan’s post hoc and by intention-to-treat analysis—delta (Δ) variation by Wilcoxon rank-sum—Mann–Whitney (data presented as median and 95% confidence interval). CTL = Control group, RET = Resistance Exercise Training group. AHI = apnea hypopnea index. min = minutes. % = percentage. n° = number of events. n°/h = number of events per hour. * difference from CTL at the same timepoint, *p* < 0.05. ^†^ difference from baseline in the same group; ^#^ difference from CTL, *p* < 0.05.

**Table 3:** Subjective sleep evaluation parameters.

Variables/Groups	CTL				RET			
	Baseline	After 12 Weeks	∆ Variation		Baseline	After 12 Weeks	∆ Variation	
			Median	95%CI			Median	95%CI
Epworth _(score)_	5.21 ± 3.09	5.57 ± 3.50	0.00	2.85–6.33	6.78 ± 4.13	6.50 ± 5.18	0.00	2.63–5.84
Insomnia Severity Index _(score)_	5.71 ± 3.89	5.78 ± 3.78	−11.00	5.24–11.64	8.71 ± 4.37	5.14 ± 3.73 *	−14.00 ^#^	5.08–11.30
Pittsburgh _(score)_	7.75 ± 5.12	4.50 ± 3.87	0.00	1.67–3.84	5.33 ± 3.21	4.33 ± 3.78	−1.50 ^#^	2.76–6.14
Pittsburgh Sleep Efficiency _(%)_	84.50 ± 11.37	81.91 ± 11.25	0.00	8.04–17.87	75.57 ± 15.37	83.57 ± 14.56 *	9.50 ^#^	9.87–21.94

Statistical analysis by Generalized Linear Model (GLM—data presented as mean ± standard deviation) with Duncan’s post hoc and by intention-to-treat analysis—delta (Δ) variation by Wilcoxon rank-sum—Mann–Whitney (data presented as median and 95% confidence interval). CTL = Control group, RET = Resistance Exercise Training group. * difference from RET at baseline, *p* < 0.05. ^#^ difference from CTL, *p* < 0.05.

**Table 4:** Evaluation of biochemical markers.

Variables/Groups	CTL				RET			
	Baseline	After 12 Weeks	∆ Variation		Baseline	After 12 Weeks	∆ Variation	
			Median	95%CI			Median	95%CI
Testosterone _(ng/dL)_	127.00 ± 211.60	130.65 ± 226.45	−2.20	10.89–27.35	329.02 ± 298.30	322.16 ± 317.70	−4.70	64.71–148.97
GH _(ng/mL)_	1.95 ± 2.42	1.11 ± 1.32	−0.29	1.26–2.80	1.21 ± 1.26	1.26 ± 1.23	−0.52	1.30–3.01
IGF 1 _(ng/mL)_	12.27 ± 28.20	14.60 ± 28.25	−0.01	6.00–13.34	7.76 ± 20.47	7.33 ± 20.46	−0.04	1.25–2.78
Cortisol _(ug/dL)_	12.29 ± 3.07	13.40 ± 2.10	1.10	2.39–5.32	10.05 ± 3.23	11.29 ± 2.65	0.20	2.87–6.38
TNF-α _(pg/mL)_	4.30 ± 0.36	4.17 ± 0.32	−0.07	0.25–0.56	4.40 ± 0.32	4.41 ± 0.31	−0.06	0.23–0.51
IL-6 _(pg/mL)_	2.88 ± 0.75	2.56 ± 0.64	−0.25	0.77–1.71	2.92 ± 1.08	3.29 ± 1.71	0.16	0.88–1.97
IL-10 _(pg/mL)_	1.96 ± 0.58	2.26 ± 0.88	0.26	0.53–1.18	2.13 ± 0.80	2.51 ± 0.99 *	0.27	0.31–0.69
IL-1 RA _(ng/mL)_	0.95 ± 0.06	0.94 ± 0.05	−0.01	0.04–0.08	0.93 ± 0.03	0.99 ± 0.10 *	0.04 ^#^	0.06–0.15

Statistical analysis by Generalized Linear Model (GLM—data presented as mean ± standard deviation) with Duncan’s post hoc and by intention-to-treat analysis—delta (Δ) variation by Wilcoxon rank-sum—Mann–Whitney (data presented as median and 95% confidence interval). CTL = Control group, RET = Resistance Exercise Training group. ng/dL = nanogram/deciliter. ug/dL = microgram/deciliter. pg/dL = picogram/deciliter. % = percentage. * difference from RET at the baseline, *p* < 0.05. ^#^ difference from CTL, *p* < 0.05.

## Supplementary Materials

The following supporting information can be downloaded at: https://www.mdpi.com/article/10.3390/ijerph192316322/s1. Figure S1: STRENGTH assessment by the text of 1 repetition maximum (1RM). The results are presented by the BoxPlot represented by the average, maximum and minimum values. * difference to baseline; # difference for 6th training week; *p* < 0.01. Table S1: Sample characterization at baseline subgroup analysis by gender. Table S2: Evaluation of sarcopenia parameters and strength subgroup analysis by gender; Table S3. Evaluation of sleep aspects through full-night polysomnography subgroup analysis by gender; Table S4. Evaluation of biochemical markers subgroup analysis by gender.
