# Supplementary material for: Resistance Training Improves Sleep and Anti-Inflammatory Parameters in Sarcopenic Older Adults: A Randomized Controlled Trial
Source: Int J Environ Res Public Health. 2022 Dec 6;19(23):16322. doi: 10.3390/ijerph192316322 (PMC9736460; doi:10.3390/ijerph192316322)
Supplement: Supplementary file 1 [file ijerph-19-16322-s001.zip › ijerph-2005906-supplementary.pdf]

Supplementary Table S1. Sample characterization at baseline subgroup analysis by gender

Variables/Groups	**CTL-male** n=3	**CTL-female** n=11	**RET-male** n=7	**RET-female** n=7
Age *(years)*	79.33 ± 3.51	73.36 ± 7.43	75.57 ± 6.13	79.28 ± 6.26
Height *(cm)*	164.00 ± 17.77	153.84 ± 9.10	172.82 ± 6.55	151.25 ±6.45
Body Mass *(kg)*	86.63 ± 9.8#	60.59 ± 10.8	77.55 ± 7.49#	57.71 ± 8.2
Body Fat *(%)*	36.86 ± 11.60	43.67 ± 4.56	28.58 ± 6.67	42.65 ± 7.71
**AMI** (kg/m^2)	6.92 ± 0.06	5.76 ± 0.60	6.36 ± 2.32	5.67 ± 0.31
Body Mass Index (kg/m^2)	32.57 ± 5.02*	25.45 ± 2.72	25.97 ± 2.22	25.10 ± 1.92
**Hemoglobin** **(g/dl)**	14.33 ± 2.20	13.60 ± 1.27	15.34 ± 0.65	13.81 ± 1.03
**Hematocrit** **(%)**	42.16 ± 7.33	40.65 ± 3.32	45.82 ± 2.16	42.06 ± 3.87
**Platelets** **(thousands/mm^3)**	194.00 ± 43.13	189.50 ± 7.77	197.00 ± 52.85	253.83 ± 60.91
**Leukocytes** **(thousands/mm^3)**	5.30 ± 1.05	4.92 ± 1.01	7.35 ± 2.20	5.71 ± 1.01
**Neutrophils** **(thousands/mm^3)**	3.19 ± 0.45	3.07 ± 1.01	4.51 ± 1.71	3.28 ± 0.96
**Eosinophils** **(thousands/mm^3)**	0.11 ± 0.08	0.04 ± 0.05	0.41 ± 0.62	0.12 ± 0.08
**Basophils** **(thousands/mm^3)**	0.05 ± 0.01	0.01 ± 0.00	0.02 ± 0.01	0.04 ± 0.02
**Typical lymphocytes** **(thousands/mm^3)**	1.60 ± 0.61	1.51 ± 0.02	1.86 ± 0.80	1.93 ± 0.46
**Total lymphocytes** **(thousands/mm^3)**	1.60 ± 0.61	1.51 ± 0.02	1.86 ± 0.80	1.93 ± 0.46
**Monocytes** **(thousands/mm^3)**	0.32 ± 0.06	0.28 ± 0.07	0.53 ± 0.23	0.34 ± 0.07
**Cholesterol** **(mg/dl)**	153.33 ± 20.03	199.50 ± 0.71	159.60 ± 14/57	210.00 ± 45.21
**HDL** **(mg/dl)**	51.00 ± 4.58	82.00 ± 11.31	48.60 ± 10.99	59.66 ± 8.43
**NO HDL** **(mg/dl)**	102.93 ± 16.19	117.50 ± 10.60	111.00 ± 18.08	150.33 ± 47.71
**LDL** **(mg/dl)**	78.66 ± 16.77	102.50 ± 14.84	89.60 ± 11.61	126.50 ± 42.30
**VLDL** **(mg/dl)**	23.66 ± 2.08	15.00 ± 4.24	21.40 ± 10.16	23.83 ± 6.61
**Triglycerides** **(mg/dl)**	119.00 ± 11.78	74.50 ± 20.50	106.00 ± 51.26	121.16 ± 32.40
**Glucose** **(mg/dl)**	103.33 ± 5.13	94.00 ± 4.24	96.20 ± 17.54	93.33 ± 6.77
**Urea** **(mg/dl)**	31.33 ± 2.08	33.50 ± 4.90	39.80 ± 9.25	41.83 ± 14.52
**Creatinine** **(mg/dl)**	0.90 ± 0.00	0.74 ± 0.03	0.94 ± 0.10	0.75 ± 0.20
**Albumin** **(g/dl)**	4.30 ± 0.26	4.40 ± 0.28	4.38 ± 0.30	4.13 ± 0.25

Statistical Analysis by Generalized Linear Model (GLM - data presented as mean ± standard de-viation) with Duncan's post hoc. CTL = Control group, RET = Resistance Exercise Training group. AMI = appendicular body mass index. g/m2= gram/square meter. Kg = kilogram/square meter. g/dL. = gram/deciliter. thousand/mm3= thousand units/cubic millimeter. mg/dL = milli-gram/deciliter. % = percentage. * different to everyone else. # male different to female $p < 0.05$.

Supplementary Table S2. - Evaluation of sarcopenia parameters and strength subgroup analysis by gender.

	**CTL**				**RET**			
Variables/Groups	Male n=3		Female n=11		Male n=7		Female n=7	
	Baseline	After 12 weeks	Baseline	After 12 Weeks	Baseline	After 12 weeks	Baseline	After 12 weeks
**Handgrip** (Kg)	27.66 ± 4.93	22.66 ± 7.02	18.82 ± 6.65	20.06 ± 3.65	26.41 ± 5.10	34.14 ± 6.10*	16.57 ± 2.50	26.77 ± 10.11*
**SPPB** (score)	10.00 ± 1.73	10.33 ± 2.08	8.63 ± 2.73	10.36 ± 2.20	10.00 ± 1.15	11.57 ± 0.78	10.00 ± 1.82	11.28 ± 1.11
**AMI** (kg/m^2)	7.13 ± 0.44	6.93 ± 0.07	6.28 ± 0.73	5.75 ± 0.63	7.51 ± 0.48	6.36 ± 2.32	5.85 ± 0.57	6.67 ± 0.31
**PT ext. Absolute** (N-M)	69.43 ± 29.21	73.66 ± 25.12	63.63 ± 38.72	70.43 ± 39.15	80.77 ± 28.37	79.90 ± 27.11	104.12 ± 28.27	108.25 ± 38.76
**PT ext. BMR** (%)	109.53 ± 29.68	110.73 ± 28.06	99.54 ± 39.98	104.11 ± 46.57	127.12 ± 38.21	127.48 ± 36.20	154.62 ± 38.64	155.50 ± 35.02
**PT flex. Absolute** (N-M)	36.13 ± 4.63	42.13 ± 11.67	30.73 ± 21.48	41.10 ± 31.28	37.90 ± 12.40	41.94 ± 12.35	45.84 ± 15.52	48.82 ± 14.38
**PT flex. BMR** (%)	56.73 ± 16.91	66.63 ± 7.45	49.50 ± 24.52	60.89 ± 38.25	61.31 ± 17.80	66.31 ± 16.64	69.61 ± 14.10	72.15 ± 16.26
**PT isometric Absolute** (N-M)	111.10 ± 38.52	108.40 ± 51.02	72.68 ± 32.44	74.70 ± 35.03	97.05 ± 28.92	82.17 ± 19.55	118.22 ± 55.23	118.22 ± 64.81
**PT isometric BMR** (%)	161.73 ± 46.38	161.13 ± 49.55	102.52 ± 36.79	114.79 ± 45.92	124.22 ± 58.82	104.04 ± 49.51	154.50 ± 58.91	167.20 ± 51.85

Statistical Analysis by Generalized Linear Model (GLM - data presented as mean ± standard deviation) with Duncan's post hoc. CTL = Control group, RET = Resistance Exercise Training group. AMI = appendicular body mass index. kg/m2= kilogram/square meter. N-M = newton meter. % = percentage. PT= Peak Torque. Ext. = knee extension. Flex = knee flexion. BMR = body mass relative. * Different to baseline and CTL by gender $p < 0.05$.

Supplementary Table S3. - Evaluation of sleep aspects through full-night polysomnography subgroup analysis by gender.

	CTL				RET			
Variables/Groups	Male n=3		Female n=11		Male n=7		Female n=7	
	Baseline	After 12 weeks	Baseline	After 12 Weeks	Baseline	After 12 weeks	Baseline	After 12 weeks
Sleep Latency *(min)*	24.7 ± 16.52	26.70 ± 20.95	20.74 ± 16.16	30.88 ± 23.88	19.48 ± 13.32	10.05 ± 5.59	32.24 ± 24.66	22.12 ± 19.62
**REM sleep latency (min)**	110.50 ± 15.75	95.83 ± 31.86	79.59 ± 47.76	104.95 ± 49.59	101.85 ± 79.13	133.64 ± 68.98	115.21 ± 72.35	100.57 ± 74.06
**Total Sleep Time (min)**	318.56 ± 123.90	326.66 ± 95.21	348.39 ± 85.19	324.72 ± 62.81	296.10 ± 49.27	285.47 ± 36.87	323.91 ± 71.32	312.25 ± 117.69
**Sleep Efficiency (%)**	66.68 ± 16.26	71.53 ± 12.58	73.81 ± 17.65	68.93 ± 93	63.98 ± 11.22	64.71 ± 14.42	70.12 ± 15.76	71.14 ± 25.31
**N1 (%)**	11.63 ± 2.50	11.70 ± 6.14	9.90 ± 4.81	9.83 ± 3.30	27.94 ± 18.54	27.30 ± 21.07	13.31 ± 8.47	17.32 ± 22.02
**N2 (%)**	33.34 ± 6.09	37.00 ± 4.47	41.16 ± 12.66	44.12 ± 11.45	30.32 ± 10.29	33.24 ± 10.03	43.90 ± 9.80	41.51 ± 4.67
**N3 (%)**	33.53 ± 6.24	28.16 ± 3.23	31.81 ± 12.87	27.70 ± 10.25	22.51 ± 9.77	28.38 ± 12.09	28.60 ± 9.52	32.12 ± 10.91
**WASO (min)**	73.40 ± 56.42	88.95 ± 34.43	81.45 ± 47.65	100.40 ±43.06	149.07 ± 51.44	154.34 ± 73.12	107.77 ± 60.84	108.07 ± 113.74
**Apnea/hour (nº/h)**	11.56 ± 5.89	14.46 ± 2.53	9.17 ± 4.27	12.97 ± 8.62	23.62 ± 17.08	11.88 ± 7.51*	10.02 ± 5.66	2.87 ± 4.44*
**AHI (nº/h)**	30.20 ± 5.23	28.16 ± 9.68	11.10 ± 11.46	11.58 ± 15.65	15.01 ± 20.32	12.05 ± 18.74	13.15 ± 10.56	6.68 ± 6.23#
**SPO2 (%)**	13.43 ± 8.05	10.36 ± 8.69	5.27 ± 10.92	5.51 ± 11.73	8.70 ± 13.73	7.14 ± 11.65	4.98 ± 5.72	2.85 ± 4.07
**Epworth (score)**	4.33 ± 3.21	6.66 ± 4.72	5.45 ± 3.17	5.27 ± 3.31	8.14 ± 4.63	10.28 ± 4.27	5.42 ± 3.35	2.71 ± 2.56
**IGI (score)**	5.66 ± 5.50	4.00 ± 3.60	5.72 ± 3.69	6.27 ± 3.84	8.28 ± 5.18	4.85 ± 4.94	9.14 ± 3.76	5.42 ± 2.37
**PSQI (score)**	6.00 ± 4.58	5.33 ± 5.77	5.18 ± 3.31	6.09 ± 3.44	6.85 ± 3.28	5.14 ± 3.07	7.28 ± 4.75	4.42 ± 3.25
**PSQI Sleep Efficiency (%)**	85.66 ± 14.01	91.00 ± 8.54	84.18 ± 11.32	80.71 ± 11.18	76.57 ± 11.04	83.42 ± 13.19	74.57 ± 19.69	83.71 ± 16.91

Statistical Analysis by Generalized Linear Model (GLM–data presented as mean ± standard deviation) with Duncan's post hoc. CTL = Control group, RET = Resistance Exercise Training group. AHI = apnea and hypopnea index. min. = minutes. % = percentage. nº = number of events. nº/h = number of events per hour. IGI = Index Gravity Insomnia. PSQI = Pittsburgh Sleep Quality Index. * different from baseline for the same sex $p < 0.05$. # different from men in the CTL at baseline $p < 0.05$.

Supplementary Table S4. Evaluation of biochemical markers subgroup analysis by gender.

	CTL				RET			
Variables/Groups	Male n=3		Female n=11		Male n=7		Female n=7	
	Baseline	After 12 weeks	Baseline	After 12 Weeks	Baseline	After 12 weeks	Baseline	After 12 weeks
Testosterone *(ng/dL)*	551 ± 76.50*	585.45 ± 73.61*	32.70 ± 14.32	29.58 ± 15.03	588.68 ± 86.01*	579.62 ± 185.0*	26.08 ± 13.10	21.80 ± 15.03
**GH** (ng/mL)	0.36 ± 0.23	0.44 ± 0.36	2.39 ± 2.58	1.29 ± 1.44	0.69 ± 0.71	1.02 ± 1.09	1.83 ± 1.45	1.63 ± 1.36
**IGF 1** (ng/mL)	27.15 ± 43.53	28.38 ± 42.97	8.21 ± 23.89	10.84 ± 24.41	2.23 ± 2.99	2.27 ± 3.44	13.30 ± 28.76	12.39 ± 28.90
**Cortisol** (ug/dL)	11.86 ± 1.80	14.30 ± 0.79	12.40 ± 3.40	12.78 ± 2.25	9.10 ± 4.30#	11.58 ± 2.65	11.00 ± 1,45	11.00 ± 2,83
**TNF-α** (pg/mL)	4.75 ± 0.36	4,59 ± 0.28	4.18 ± 0.26	4.05 ± 0.24	4.47 ± 0.19	4.28 ± 0.22^{+}	4.33 ± 0.41	4.54 ± 0.34^{++}
**IL-6** (pg/mL)	2.71 ± 0.68	3.50 ± 0.33	2.92 ± 0.80	2.30 ± 0.41	2.76 ± 1.25	3.15 ± 0.96	3.09 ± 0.95	3.44 ± 2.32
**IL-10** (pg/mL)	2.28 ± 0.82	3.28 ± 0.89^	1.88 ± 0.52	1.98 ± 0.68	2.11 ± 0.63	2.30 ± 0.84	2.14 ± 1.00	2.72 ± 1.14
**IL-1 RA** (ng/mL)	1.01 ± 0.09	0.98 ± 0.04	0.93 ± 0.05	0.93 ± 0.05	0.94 ± 0.04	1.01 ± 0.14‡	0.92 ± 0.03	0.97 ± 0.05

Statistical Analysis by Generalized Linear Model (GLM - data presented as mean ± standard deviation) with Duncan's *post hoc*. CTL = Control group, RET = Resistance Exercise Training group. ng/dL. = nanogram/deciliter. ug/dL. = microgram/deciliter. pg/dL = picogram/deciliter. % = percentage. *different to female at same timepoint; $p< 0.05$. # different to CTL-male at 12weeks; $p< 0.05$. $^{+}$different to CTL-male at same time-point; $p< 0.05$. $^{++}$different to CTL-female at same time-point; $p< 0.05$. ^different to all; $p< 0.05$. ‡ different to RET-male at baseline.

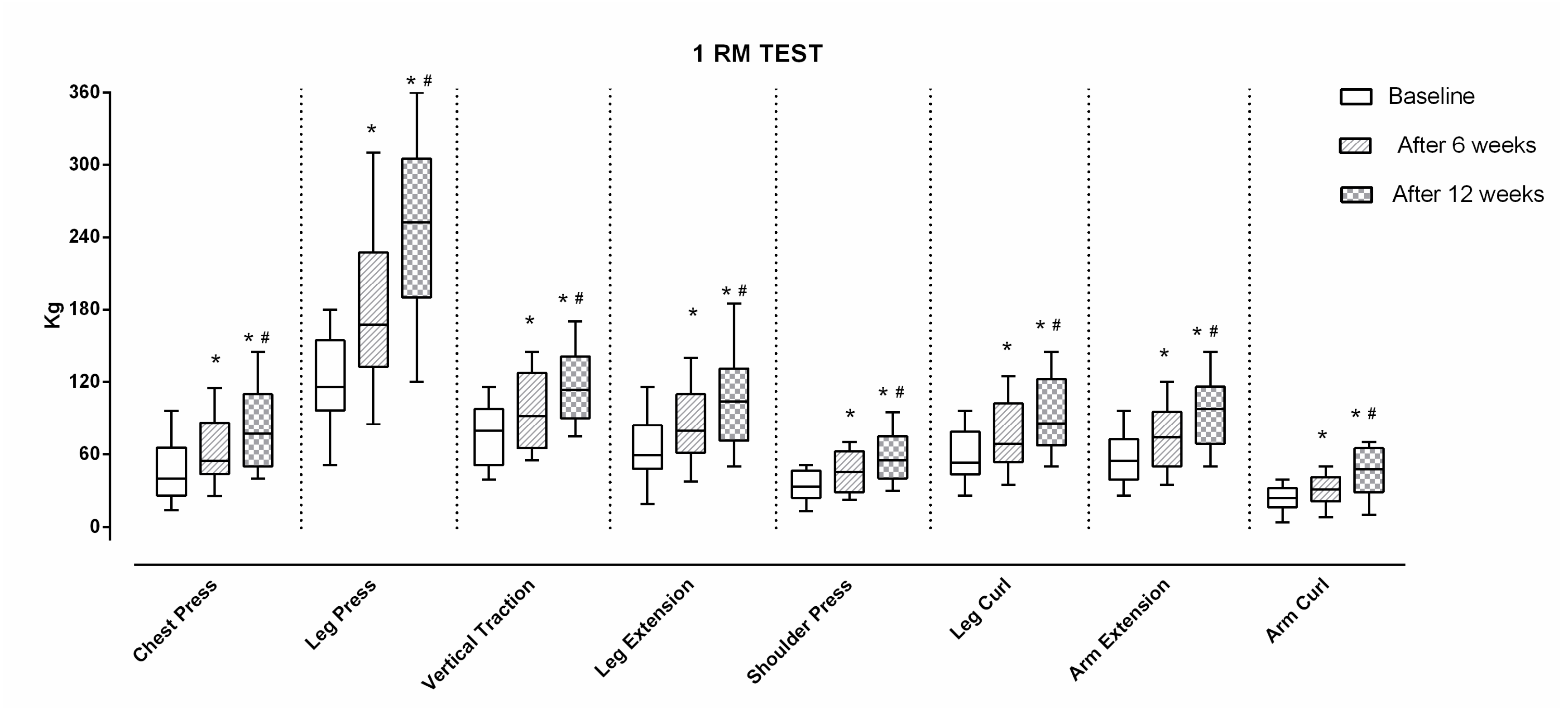

Figure S1: STRENGTH assessment by the text of 1 repetition maximum (1RM). The results are presented by the BoxPlot represented by the average, maximum and minimum values. * difference to baseline; # difference for 6th training week; $p < 0.01$
